# Phase angle as a predictor of nutritional status and quality of life in hemodialysis patients

**DOI:** 10.1371/journal.pone.0348498

**Published:** 2026-05-14

**Authors:** Elif İradeli, Sema Çalapkorur

**Affiliations:** 1 Department of Nutrition and Dietetics, Malatya Turgut Özal University Faculty of Health Science, Malatya, Türkiye; 2 Department of Nutrition and Dietetics, Erciyes University Faculty of Health Science, Kayseri, Türkiye; Instituto Politecnico de Santarem Escola Superior de Desporto de Rio Maior, PORTUGAL

## Abstract

**Background and objective:**

Hemodialysis (HD) patients are at high risk for protein-energy wasting and cellular membrane deterioration, which significantly impact their clinical outcomes. Phase angle (PhA) is an objective parameter for assessing nutritional status. As PhA is an indicator of physical function and cellular health, it is hypothesized to correlate with Quality of Life (QoL). This study was carried out to determine the relationship between the PhA and the nutritional status and QoL in patients receiving HD treatment.

**Methods:**

This cross-sectional study was conducted with 98 patients. Nutritional Risk Score-2002 (NRS-2002) and Malnutrition Inflammation Score (MIS) scales were used to assess the nutritional status of the patients. Physical activity level and QoL were determined by the Physical Activity Questionnaire-Short Form (IPAQ-SF) and Kidney Disease Quality of Life Short Form-36 (KDQOL^TM^-36), respectively. The body composition of the patients was measured by Inbody S10 and the PhA value was also obtained from this measurement.

**Results:**

The mean PhA value of the patients was determined as 5.1˚ ± 1.1˚. As a result of the ROC analysis performed using the NRS-2002, the PhA cutoff point was calculated as 4.85˚. It was observed that patients with a PhA less than 4.85˚ had higher MIS and NRS-2002 scores than patients with a PhA greater than this value. KDQOL^TM^-36 physical and mental component sub-dimension scores of patients with PhA greater than 4.85˚ were found to be higher than those of patients with a PhA less than 4.85˚. It has also been determined that patients with a PhA less than 4.85˚ have a more sedentary lifestyle than patients with a PhA greater than this value.

**Conclusions:**

PhA is a useful parameter in determining nutritional status and QoL in HD patients. Therefore, PhA may serve as a supportive parameter for evaluating the nutritional and functional status of HD patients in clinical settings.

## 1. Introduction

Chronic kidney disease (CKD) is defined as a syndrome characterized by structural or functional renal impairment [1]. Due to its high morbidity and mortality risks, CKD remains a significant public health challenge [2]. For patients with end-stage renal disease, renal replacement therapies such as hemodialysis (HD) are essential for survival [3]. Despite technological advancements, malnutrition remains highly prevalent in the HD population [4] and is directly associated with adverse clinical outcomes, necessitating early and precise nutritional monitoring [5].

As no single “gold standard” exists for determining nutritional status, a multi-parameter assessment is generally recommended [6]. Bioelectrical impedance analysis (BIA) has emerged as a key tool in this context [7]. Phase angle (PhA), a parameter derived from BIA [8], reflects cellularity, membrane integrity, and vital cell function [9]. Conversely, a lower PhA indicates cellular depletion or impaired membrane function [10]. Beyond nutritional screening, PhA serves as a prognostic indicator [11] and is a sensitive marker for malnutrition in HD patients [12–14]. Furthermore, lower PhA values have been shown to significantly increase mortality rates in this population [15].

The biological status reflected by PhA is closely linked to a patient’s well-being. Factors such as chronic inflammation and malnutrition, which decrease PhA, also lead to a decline in Quality of Life (QoL) and functional capacity [9,16]. Deteriorating nutritional status is a primary driver for the reduced QoL observed in long-term dialysis patients [17]. Conceptually, PhA serves as an objective indicator of cellular health, which fundamentally underpins the physical and functional domains of a patient's QoL. However, the clinical utility of PhA is currently hindered by a lack of consensus regarding standardized cut-off points [18–20]. This study, therefore, aims to investigate the relationship between PhA, nutritional status, and QoL in HD patients, while also calculating a study-specific cut-off point to contribute to clinical standardization. It is hypothesized that higher PhA values are positively associated with better nutritional status and higher QoL scores, and that the study-specific PhA cut-off point serves as a significant predictor for identifying malnutrition risk in HD patients.

## 2. Methods

### 2.1. Study design and population

This cross-sectional study was conducted between November 2021 and February 2022 at a university hospital dialysis unit in Türkiye.

#### 2.1.1. Eligibility criteria.

The study population was selected based on the following criteria:

- **Inclusion Criteria:** Patients aged >18 years, receiving HD treatment three times a week for at least three months, and being clinically stable.
- **Exclusion Criteria:** Presence of a permanent pacemaker or implantable cardioverter-defibrillator, acute inflammation (CRP > 10 mg/L) or active bleeding, limb amputations, history of renal transplantation, malignant tumors, and pregnancy.

#### 2.1.2. Sample size and recruitment.

**Fig 1:**
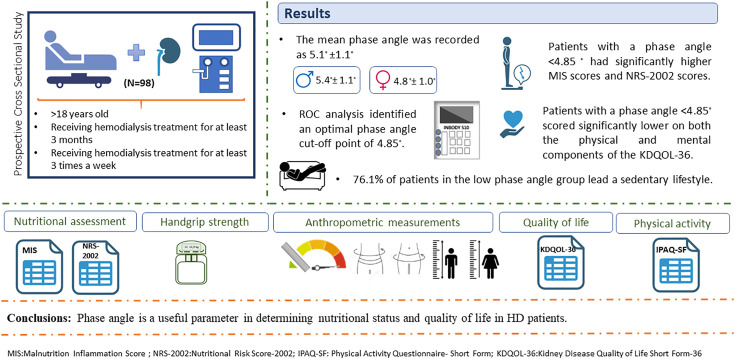
Graphical abstract of the study.

The sample size was determined using G*Power software (version 3.1.9.2). An ‘a priori’ power analysis was conducted within the ‘Exact’ test family using the ‘Correlation: Bivariate normal model’ statistical test. Based on a moderate effect size (ρ = 0.3), a significance level (alpha) of 0.05, and a statistical power (1-β) of 0.80, the minimum required sample size was calculated as N = 82. To account for potential drop-outs or missing data, intentional oversampling was performed, and the study was completed with 98 patients. The recruitment process and patient flow are detailed in the Participant Flow Diagram (Fig 1).

### 2.2. Ethical standards and data management

The study was conducted in strict accordance with the ethical principles for medical research involving human subjects outlined in the Declaration of Helsinki. Approval was granted by the Erciyes University Clinical Research Ethics Committee (Decision No: 2021/441). Prior to data collection, all participants were provided with a comprehensive verbal explanation of the study objectives, the voluntary nature of participation, and the strict anonymity of their data. It was explicitly clarified that participants maintained the right to withdraw from the study at any point without any impact on their dialysis treatment. Following a sufficient period for questions and consideration, written informed consent was obtained from all volunteers, covering both participation and the publication of research findings.

### 2.3. Data collection and clinical protocols

To ensure inter-observer reliability and minimize bias, all anthropometric measurements, hand-grip tests, and BIA procedures were performed by the same trained researcher.

#### 2.3.1. Bioelectrical impedance analysis.

Body composition and PhA values (whole body, trunk, and extremities) were measured using the InBody S10 (InBody Medikal, Istanbul, Türkiye). Measurements were performed within 30 minutes following the HD session. This timing was selected to ensure the patient had reached their “dry weight” and to allow for the stabilization of fluid redistribution, which is essential for the accuracy of post-dialysis BIA in HD populations. The Inbody S10 is a segmental multifrequency device capable of measuring the trunk and each extremity separately for body composition assessment. Care was taken to ensure that patients did not have any metal objects on them during the measurement. The analysis was performed with the patient lying down, legs apart, and arms not touching the torso. Eight electrodes were placed on the thumbs and middle fingers of both hands and on the ankles of the patients according to the device's instructions for use.

#### 2.3.2. Anthropometric and hand-grip strength measurements.

- **Anthropometry:** The anthropometric measurements performed as part of this study were taken following a HD session. The patients wore light clothing and were barefoot during the measurements. Body weight (dry weight, kg) was measured using a 0.1 kg sensitive calibrated medical-grade digital scale (**Seca 813, Hamburg, Germany**). Height was measured to the nearest 0.1 cm using a portable standard wall-mounted stadiometer (**Seca 206, Hamburg, Germany**), with participants standing straight, head aligned according to the Frankfurt plane, arms hanging freely at their sides, feet together and with heels, buttocks and shoulder blades in contact with the vertical surface of the stadiometer. The body mass index value was calculated using the formula [dry weight(kg)/length(m)^2^] from the obtained dry weight and length values. Waist circumference measurement was taken with the patient standing, abdomen relaxed, arms at the sides, and feet together; at the midpoint between the lowest rib and the iliac crest, using a non-stretchable measuring tape. Hip circumference was measured at its widest point while the patient was standing with arms at their sides and feet together [21]. Triceps skinfold thickness (TST) was measured from the arm without an arteriovenous fistula to the nearest 0.1 mm, using a Holtain skinfold caliper. Mid-arm circumference (MAC) was measured at the acromion-olecranon midpoint using a tape measure on the non-fistula arm. Mid-upper arm circumference (MAC), waist, and hip circumferences were measured using a non-elastic tape. Mid-upper arm muscle circumference (MAMC) was calculated using the equation MAC (cm) − π × TST (cm).
- **Hand-grip Strength (HGS):** Measured from the arteriovenous fistula arm using a Camry Digital Handgrip Dynamometer (Model EH101) after the dialysis session. The aim was to minimize potential calibration errors by using the newly acquired dynamometer for this study. The measurements were performed using the protocol recommended by the American Society of Hand Therapists. Measurements were taken with HD patients in the following positions: shoulder adduction, elbow 90° flexion, wrist 30° extension, and 0–15° ulnar deviation [22]. Three consecutive measurements were taken at 1 minute intervals, and the arithmetic mean (kg) was recorded.

#### 2.3.3. Questionnaires and nutritional screening.

- **General Questionnaire:** A researcher-administered form was used to collect sociodemographic and clinical data. The questions were designed based on a comprehensive literature review of the HD population to ensure content and face validity.
- **Nutritional Risk Screening (NRS-2002):** Used to identify nutritional risk; a total score 3 indicates risk.
- **Malnutrition Inflammation Score (MIS):** A validated tool for HD patients consisting of medical history, physical examination, BMI, and laboratory parameters. Higher scores indicate more severe malnutrition and inflammation.
- **IPAQ-SF:** Used to determine physical activity levels based on MET-min/week.
- **KDQOL-36:** A disease-specific tool used to assess health-related quality of life across three dimensions: symptoms, burden, and effects of kidney disease.
- **Biochemical Data:** The biochemical data (Albumin and TIBC) were retrieved from the patients’ medical records and corresponded to the routine laboratory tests performed at the time of the BIA assessment.

### 2.4. Statistical analysis

Data were analyzed using IBM SPSS Statistics for Windows, Version 22.0 (IBM Corp., Armonk, NY, USA). Normality was assessed via the Shapiro-Wilk test. Categorical variables were compared using Chi-square tests. For continuous variables, independent T-tests or Mann-Whitney U tests were used based on distribution. Effect sizes for pairwise comparisons were calculated using Cohen’s $d$ and interpreted as small (0.20), medium (0.50), or large (0.80). Relationships were examined using Pearson or Spearman correlation coefficients. The strength of these correlations (r) was evaluated as weak (0.00–0.29), moderate (0.30–0.49), strong (0.50–0.69), or very strong (0.70–1.00). ROC analysis was performed to determine optimal PhA cut-off points. Partial correlation coefficients (*pr*) were calculated to assess the relationships between phase angle and nutritional/biochemical parameters, adjusting for age, sex, diabetes mellitus, hypertension, and cardiovascular disease. Data were analyzed using IBM SPSS Statistics for Windows, Version 22.0 (IBM Corp., Armonk, NY, USA). A p-value <0.05 was considered statistically significant.

## 3. Results

### 3.1. Baseline characteristics and malnutrition prevalence

The study was conducted with 98 patients (41.8% male, 58.2% female) with a mean age of 55.51 ± 11.96 years. As illustrated in Table 1, the prevalence of malnutrition risk varied according to the screening tool utilized. According to the MIS, 67.3% of the total cohort exhibited malnutrition risk, with a higher prevalence observed in females compared to males (75.4% vs. 56.1%; p = 0.073). In contrast, the NRS-2002 identified a lower overall malnutrition risk of 16.7% for the entire study population.

**Table 1:** Distribution of malnutrition risk status of participants by gender.

	Male (n = 41)		Female (n = 57)		Total (n = 98)	
	N	%	N	%	N	%
**MIS ≤ 5** **No Risk of Malnutrition**	18	43.9	14	25.6	32	32.7
**MIS > 5** **Risk of Malnutrition**	23	56.1	43	75.4	66	67.3
	p(^a)^ =0.073 x^2^ =3.225					
	**Male (n = 14)**		**Female (n = 34)**		**Total (n = 48)**	
	**N**	**%**	**N**	**%**	**N**	**%**
**NRS-2002 < 3 points** **No Risk of Malnutrition**	12	85.7	28	82.4	40	83.3
**NRS-2002 ≥ 3 points** **Risk of Malnutrition**	2	14.3	6	17.6	8	16.7
	p^(b)^ =1.000 x^2^ = 0.000					

MIS = Malnutrition inflammation score, NRS-2002 = Nutritional risk score. Chi-square test with Yates correction (a); Fisher's exact test (b) were performed.

### 3.2. Diagnostic performance of phase angle

**Fig 2:**
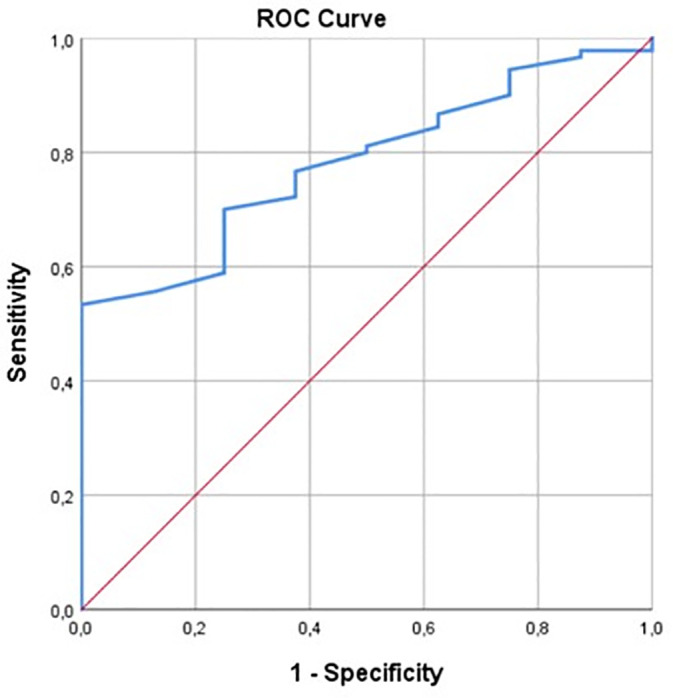
ROC curve of Phase Angle (PhA) for predicting nutritional risk according to NRS-2002.

Sexual dimorphism was evident in PhA values, as male patients exhibited significantly higher mean PhA compared to females (5.4º ± 1.1º vs. 4.8º ± 1.0º; p < 0.05). The mean PhA for the entire cohort was recorded as 5.1º ± 1.1º. To evaluate the efficacy of PhA as a nutritional biomarker, a ROC analysis was performed using NRS-2002 as the reference standard. The analysis identified an optimal PhA cut-off point of 4.85º. This threshold demonstrated a robust Area Under the Curve (AUC) of 0.782 (p = 0.008; 95% CI: 0.659–0.905), indicating substantial diagnostic accuracy. The sensitivity and specificity for this cut-off were obtained as 0.556 and 0.875, respectively (Table 2) (Fig 2).

**Table 2:** Results of the ROC analysis.

	AUC	Standard error	Sensitivity	Specificity	95% Confidence Interval		p
					Lower limit	Upper limit	
NRS-2002	0.782	0.063	0.556	0.875	0.659	0.905	**0.008**

NRS-2002 = Nutritional risk score; AUC: Area Under the Curve.

### 3.3. Comparison of clinical outcomes by PhA threshold

When participants were categorized based on the 4.85º threshold, significant differences were revealed in nutritional and clinical outcomes (Table 3). Patients with a PhA below the cut-off (<4.85º) exhibited significantly higher MIS scores (7.1 ± 3.0 vs. 6.0 ± 2.5; p = 0.044, Cohen’s d = 0.40) and higher NRS-2002 scores (2.0 ± 0.7 vs. 1.6 ± 0.7; p = 0.040, Cohen’s d = 0.57), reflecting a more severe state of malnutrition and inflammation. Furthermore, lower PhA levels were associated with a decline in health-related quality of life. As shown in Table 4, patients below the 4.85º threshold recorded significantly lower scores in both the Physical Component (33.9 ± 10.4 vs. 39.1 ± 11.0; p = 0.018, Cohen’s d = 0.49) and Mental Component (37.6 ± 12.9 vs. 42.2 ± 10.2; p = 0.037, Cohen’s d = 0.39) of the KDQOL-36. Physical activity levels also differed significantly; while 76.1% of patients in the low PhA group led a sedentary lifestyle, nearly half of those above the threshold achieved moderate physical activity levels (p = 0.009; Table 5).

**Table 3:** MIS and NRS-2002 screening test scores by phase angle cutoff point.

	Phase Angle <4.85 ˚		Phase Angle >4.85˚	
	$\overset{―}{𝐱}$ ± SS	Median/(Min-Max)	$\overset{―}{𝐱}$ ± SS	Median/(Min-Max)
**MIS**	7.1 ± 3.0	7.0/(0.0-14.0)	6.0 ± 2.5	6.0/(0.0-12.0)
	**p**^**(a)**^**:0.044** t:2.046			
**NRS-2002**	2.0 ± 0.7	2.0/(1.0-3.0)	1.6 ± 0.7	2.0/(1.0-4.0)
	**p**^**(b)**^**:0.040** Z:-2.051			

MIS = malnutrition inflammation score, NRS-2002 = nutritional risk score-2002. t test (a), Mann Whitney U (b) were performed.

**Table 4:** Distribution of participants’ KDQOL™-36 scale scores according to phase angle cutoff point.

	Phase Angle<4.85 ˚		Phase Angle>4.85˚	
	$\overset{―}{𝐱}$ ± SS	Median (Min-Max)	$\overset{―}{𝐱}$ ± SS	Median (Min-Max)
**Physical Component**	33.9 ± 10.4	34.1 (11.4-54.9)	39.1 ± 11.0	40.8 (18.0-57.9)
	**p** ^**(a)**^ **:0.018 t:-2.410**			
**Mental Component**	37.6 ± 12.9	35.5 (18.2-62.3)	42.2 ± 10.2	42.0 (20.9-62.1)
	**p** ^**(b)**^ **:0.037 Z:-2.089**			
**The Burden of Kidney Disease**	37.2 ± 30.5	25.0 (0.0-100.0)	42.1 ± 29.0	31.3 (0.0-100.0)
	p^(b)^:0.263 Z:-1.118			
**Symptoms and Problems**	71.4 ± 17.3	75.0 (25.0-100.0)	67.0 ± 20.1	66.7 (14.6-97.9)
	p^(b)^:0.325 Z: −0.983			
**The Impact of Kidney Disease on Life**	61.1 ± 25.4	68.8 (0.0-93.8)	63.8 ± 22.7	65.6 (21.9-100.0)
	p^(b)^:0.892 Z:-0.135			

t test (a), Mann Whitney U (**) were performed.

**Table 5:** Classification of participants’ physical activity level according to phase angle.

	Phase Angle <4.85˚		Phase Angle>4.85˚	
	S	%	S	%
**Category-1 (low-sedentary)**	35	76.1	25	48.1
**Category-2 (medium)**	10	21.7	25	48.1
**Category-3 (high)**	1	2.2	2	3.8
	**p=0.009, x**^**2**^ **=8.162**			

Fisher's exact test was performed.

### 3.4. Correlation analyses

The interrelationships between PhA and various clinical parameters were explored through correlation analysis (Table 6). PhA showed moderate significant positive correlations with skeletal muscle weight (r = 0.450, p < 0.001), and weak positive correlations with hand-grip strength (r = 0.290, p = 0.014) and serum TIBC (r = 0.290, p = 0.013). Notably, a very strong inverse relationship was observed between PhA and the extracellular water (ECW) ratio (r = −0.879, p < 0.001), emphasizing its sensitivity to cellular integrity and hydration status. Additionally, moderate negative correlations were found between PhA and longer dialysis vintage (r = −0.342, p = 0.003) and higher MIS scores (r = −0.314, p = 0.007).

**Table 6:** Partial correlation analysis between phase angle and various variables.

	Phase Angle	
	*pr*	p
Body weight	**0.275**	**0.019**
BMI (kg/m^2^)	**0.278**	**0.018**
Waist circumference (cm)	**0.246**	**0.037**
MAC (cm)	**0.328**	**0.005**
MAMC (cm)	**0.270**	**0.022**
TST (mm)	**0.399**	**0.001**
Body Fat Weight (kg)	0.178	0.135
Skeletal Muscle Weight (kg)	**0.450**	**< 0.001**
Total Body Water	**0.345**	**0.003**
Extracellular Water Ratio	**−0.879**	**< 0.001**
Albumin (g/dl)	0.056	0.641
TIBC	**0.290**	**0.013**
Energy intake (kkal/kg/day)	−0.123	0.304
Protein intake (g/kg/day)	−0.127	0.286
Carbohydrate intake (g/kg/day)	−0.071	0.555
Fat intake (g/kg/day)	−0.146	0.220
Dialysis vintage (years)	**−0.342**	**0.003**
NRS-2002	−0.132	0.432
MIS	**−0.314**	**0.007**
Handgrip strength	**0.290**	**0.014**

BMI = Body mass index, MAC = Mid-upper arm circumference, MAMC = Mid-upper arm muscle circumference, TST = Triceps skinfold thickness, TIBC = Total iron binding capacity, MIS = Malnutrition inflammation score, NRS-2002 = Nutritional risk score-2002, DM = Diabetes mellitus, HT = Hypertension, CVD = Cardiovascular disease.

Adjusted for age, sex, diabetes mellitus, hypertension and cardiovascular disease.

## 4. Discussion

Malnutrition remains a prevalent and critical complication in HD patients, necessitating early detection to facilitate timely interventions. BIA has emerged as a practical and non-invasive tool for evaluating body composition in this population. Specifically, the PhA derived from BIA serves as a proxy for cell membrane integrity and cellular vitality, making it a promising parameter for prognostic and nutritional assessment [23]. In our study results of the ROC analysis showed that PhA had moderate sensitivity (0.556) in diagnosing malnutrition. This finding indicates that using only a PhA value of 4.85 º for the diagnosis of malnutrition in HD patients is inadequacy. The development of malnutrition in HD patients involves many different factors, and we recommend the use of screening tools in combination rather than a single tool to assess nutritional status in this patient group.

### 4.1. Demographic influences and phase angle thresholds

Cellular health and PhA values are significantly influenced by age, biological sex, fluid distribution, and BMI [11]. In the current study, we observed a mean PhA of 5.1º ± 1.1º, with significantly higher values in male patients compared to females (5.4º ± 1.1º vs. 4.8º ± 1.0º). This sexual dimorphism is consistent with literature indicating that women typically possess lower lean body mass, resulting in reduced reactance values and subsequently lower PhA [24].

Using the NRS-2002 as a reference standard, we identified a PhA cut-off point of 4.85ºfor this cohort. When patients were stratified by this threshold, those below 4.85º exhibited significantly higher nutritional risk scores. While this categorization demonstrates the internal consistency of PhA as a marker within our sample, it is important to acknowledge that this threshold was derived from our specific study population. Nevertheless, our partial correlation analysis revealed an inverse correlation between PhA and the MIS, reinforcing findings from previous studies [25,26]. Interestingly, our study is among the first to explore and confirm a significant relationship between PhA and NRS-2002 scores in HD patients, suggesting that PhA could complement traditional screening tools.

### 4.2. Quality of life and physical activity

Malnutrition profoundly impacts the prognosis and QoL in chronic kidney disease patients [27]. We observed that patients with a PhA > 4.85º recorded significantly higher scores in both physical and mental QoL sub-dimensions. The physical component is often the most severely affected dimension in end-stage kidney disease [28,29]. Our results suggest that as PhA increases-reflecting better cellular integrity-physical functioning and general well-being improve, aligning with prior reports [25,30,31].

Furthermore, physical activity appears to be intrinsically linked to PhA values. In our cohort, a sedentary lifestyle was recorded in 76.1% of patients below the PhA threshold, whereas those with higher PhA exhibited moderate-to-high activity levels. Mechanistically, physical activity enhances muscle mass and cell membrane integrity, which increases reactance and decreases resistance [32]. Consequently, PhA may serve as a sensitive indicator of the positive effects of exercise on cellular health in HD patients [33].

### 4.3. Muscle mass, functional status, and hydration dynamics

A primary indicator of malnutrition in HD patients is the loss of muscle mass, which is strongly associated with adverse outcomes [34]. We revealed an inverse correlation between PhA and dialysis vintage, while positive correlations were observed between PhA and muscle-related markers, including MAMC, skeletal muscle weight, and hand-grip strength (HGS). HGS is a critical determinant of functional status in CKD [35]. Our findings parallel existing literature confirming that PhA is a reliable reflector of functional muscle strength [20,25].

However, a nuanced interpretation of BIA in HD is required regarding hydration and inflammation. Although PhA is often considered more resistant to fluid fluctuations than raw resistance or reactance values, it is not entirely independent of hydration status [36]. Our results suggest that PhA is sensitive to fluid redistribution, evidenced by the strong inverse correlation (r = −0.879) between PhA and ECW ratio. Given that our measurements were taken 30 minutes post-dialysis—a period of significant fluid shift-this sensitivity must be factored into clinical assessments, rather than assuming total independence from hydration status. The ECW ratio is a value calculated by Inbody S10 using the formula ECW/ total body water. The ECW ratio is recommended for assessing volume status in HD patients because excess fluid accumulates primarily in the extracellular compartment [37]. In addition, studies have shown that an increased ECW ratio is associated with malnutrition in patients with HD [38,39]. In our study, the negative correlation between the ECW ratio and the PhA supports this finding.

### 4.4. Study limitations

This study has several limitations that warrant consideration. First, the cross-sectional design limits our ability to establish causal relationships between the variables as the data represents a single point in time. Second, while data collection occurred during the COVID-19 pandemic-a period associated with increased psychological stress—this is not considered a primary weakness of the study, as the sample size was statistically sufficient according to the power analysis to ensure robust results. Furthermore, to address potential measurement interference from skin dryness, we implemented a rigorous standardized protocol involving skin preparation and moisturization at the electrode interface. This ensured reliable current conduction and minimized contact impedance. Therefore, while these environmental and physiological factors were present, they were systematically mitigated to protect the integrity of the BIA-derived parameters.

## 5. Conclusions

The findings of this study suggest that the PhA is a valuable indicator associated with nutritional status, health-related quality of life, and physical activity levels in HD patients. Monitoring PhA values may facilitate the early identification of deteriorations in nutritional status, potentially allowing for timely clinical interventions. Given its practical application, PhA should be considered a complementary biomarker alongside traditional tools for the routine evaluation of HD patients in clinical settings. While the current lack of standardized reference values for PhA in the HD population remains a challenge, the findings of this study contribute to the growing body of evidence regarding its clinical utility. The cut-off point identified in this cohort (4.85º) provides a preliminary reference for this specific population; however, further multi-center studies with larger sample sizes are necessitated to validate these results and establish universal reference standards.

## References

[1] Romagnani P, Remuzzi G, Glassock R, Levin A, Jager KJ, Tonelli M, et al. Chronic kidney disease. Nat Rev Dis Primers. 2017;3:17088. doi: 10.1038/nrdp.2017.88 29168475

[2] Pawlaczyk W, Rogowski L, Kowalska J, Stefańska M, Gołębiowski T, Mazanowska O, et al. Assessment of the Nutritional Status and Quality of Life in Chronic Kidney Disease and Kidney Transplant Patients: A Comparative Analysis. Nutrients. 2022;14(22):4814. doi: 10.3390/nu14224814 36432502 PMC9692759

[3] Kalantar-Zadeh K, Jafar TH, Nitsch D, Neuen BL, Perkovic V. Chronic kidney disease. Lancet. 2021;398(10302):786–802. doi: 10.1016/S0140-6736(21)00519-5 34175022

[4] Sahathevan S, Khor B-H, Ng H-M, Gafor AHA, Mat Daud ZA, Mafra D, et al. Understanding Development of Malnutrition in Hemodialysis Patients: A Narrative Review. Nutrients. 2020;12(10):3147. doi: 10.3390/nu12103147 33076282 PMC7602515

[5] Majlessi A, Burton JO, March DS. The effect of extended hemodialysis on nutritional parameters: a systematic review. J Nephrol. 2022;35(8):1985–99. doi: 10.1007/s40620-022-01395-w 35960430 PMC9584983

[6] Reber E, Gomes F, Vasiloglou MF, Schuetz P, Stanga Z. Nutritional risk screening and assessment. J Clin Med. 2019;8(7):1065. doi: 10.3390/jcm8071065 31330781 PMC6679209

[7] Marra M, Sammarco R, De Lorenzo A, Iellamo F, Siervo M, Pietrobelli A, et al. Assessment of Body Composition in Health and Disease Using Bioelectrical Impedance Analysis (BIA) and Dual Energy X-Ray Absorptiometry (DXA): A Critical Overview. Contrast Media Mol Imaging. 2019;2019:3548284. doi: 10.1155/2019/3548284 31275083 PMC6560329

[8] Bosy-Westphal A, Danielzik S, Dörhöfer R-P, Later W, Wiese S, Müller MJ. Phase angle from bioelectrical impedance analysis: population reference values by age, sex, and body mass index. JPEN J Parenter Enteral Nutr. 2006;30(4):309–16. doi: 10.1177/0148607106030004309 16804128

[9] Di Vincenzo O, Marra M, Di Gregorio A, Pasanisi F, Scalfi L. Bioelectrical impedance analysis (BIA) -derived phase angle in sarcopenia: A systematic review. Clin Nutr. 2021;40(5):3052–61. doi: 10.1016/j.clnu.2020.10.048 33183880

[10] Stobäus N, Pirlich M, Valentini L, Schulzke JD, Norman K. Determinants of bioelectrical phase angle in disease. Br J Nutr. 2012;107(8):1217–20. doi: 10.1017/S0007114511004028 22309898

[11] Lukaski HC, Kyle UG, Kondrup J. Assessment of adult malnutrition and prognosis with bioelectrical impedance analysis: phase angle and impedance ratio. Curr Opin Clin Nutr Metab Care. 2017;20(5):330–9. doi: 10.1097/MCO.0000000000000387 28548972

[12] Zhang G, Huo X, Wu C, Zhang C, Duan Z. A bioelectrical impedance phase angle measuring system for assessment of nutritional status. Biomed Mater Eng. 2014;24(6):3657–64. doi: 10.3233/BME-141193 25227080

[13] Lee JE, Jo IY, Lee SM, Kim WJ, Choi HY, Ha SK, et al. Comparison of hydration and nutritional status between young and elderly hemodialysis patients through bioimpedance analysis. Clin Interv Aging. 2015;10:1327–34. doi: 10.2147/CIA.S86229 26316728 PMC4541557

[14] Rimsevicius L, Gincaite A, Vicka V, Sukackiene D, Pavinic J, Miglinas M. Malnutrition Assessment in Hemodialysis Patients: Role of Bioelectrical Impedance Analysis Phase Angle. J Ren Nutr. 2016;26(6):391–5. doi: 10.1053/j.jrn.2016.05.004 27450205

[15] Pupim LB, Caglar K, Hakim RM, Shyr Y, Ikizler TA. Uremic malnutrition is a predictor of death independent of inflammatory status. Kidney Int. 2004;66(5):2054–60. doi: 10.1111/j.1523-1755.2004.00978.x 15496179

[16] Moledina DG, Perry Wilson F. Pharmacologic Treatment of Common Symptoms in Dialysis Patients: A Narrative Review. Semin Dial. 2015;28(4):377–83. doi: 10.1111/sdi.12378 25913502

[17] Bakaloudi DR, Siargkas A, Poulia KA, Dounousi E, Chourdakis M. The Effect of Exercise on Nutritional Status and Body Composition in Hemodialysis: A Systematic Review. Nutrients. 2020;12(10):3071. doi: 10.3390/nu12103071 33050111 PMC7601723

[18] Karavetian M, Salhab N, Rizk R, Poulia KA. Malnutrition-Inflammation Score VS Phase Angle in the Era of GLIM Criteria: A Cross-Sectional Study among Hemodialysis Patients in UAE. Nutrients. 2019;11(11):2771. doi: 10.3390/nu11112771 31739568 PMC6893836

[19] Lim C-K-M, Lim J-H, Ibrahim I, Chan Y-M, Zakaria NF, Yahya R, et al. Bioelectrical Impedance Analysis Derived-Phase Angle as a Pragmatic Tool to Detect Protein Energy Wasting among Multi-Ethnic Hemodialysis Patients. Diagnostics (Basel). 2021;11(10):1745. doi: 10.3390/diagnostics11101745 34679443 PMC8534349

[20] Tan R-S, Liang D-H, Liu Y, Zhong X-S, Zhang D-S, Ma J. Bioelectrical Impedance Analysis-Derived Phase Angle Predicts Protein-Energy Wasting in Maintenance Hemodialysis Patients. J Ren Nutr. 2019;29(4):295–301. doi: 10.1053/j.jrn.2018.09.001 30446269

[21] Lohman TJ, Roache AF, Martorell R. Anthropometric standardization reference manual. Champaign: Human Kinetic Books; 1988.

[22] Bakkal H, Dizdar OS, Erdem S, Kulakoğlu S, Akcakaya B, Katırcılar Y, et al. The Relationship Between Hand Grip Strength and Nutritional Status Determined by Malnutrition Inflammation Score and Biochemical Parameters in Hemodialysis Patients. J Ren Nutr. 2020;30(6):548–55. doi: 10.1053/j.jrn.2020.01.026 32197719

[23] Çalapkorur S, İradeli E. A new method for evaluating nutritional status in hemodialysis patients: phase angle. Institute Health Sci J. 2023;8(1):58–64. doi: 10.51754/cusbed.1136058

[24] Cancello R, Brunani A, Brenna E, Soranna D, Bertoli S, Zambon A, et al. Phase angle (PhA) in overweight and obesity: evidence of applicability from diagnosis to weight changes in obesity treatment. Rev Endocr Metab Disord. 2023;24(3):451–64. doi: 10.1007/s11154-022-09774-1 36484943 PMC9735068

[25] Beberashvili I, Azar A, Sinuani I, Shapiro G, Feldman L, Stav K, et al. Bioimpedance phase angle predicts muscle function, quality of life and clinical outcome in maintenance hemodialysis patients. Eur J Clin Nutr. 2014;68(6):683–9. doi: 10.1038/ejcn.2014.67 24736681

[26] Santin F, Rodrigues J, Brito FB, Avesani CM. Performance of subjective global assessment and malnutrition inflammation score for monitoring the nutritional status of older adults on hemodialysis. Clin Nutr. 2018;37(2):604–11. doi: 10.1016/j.clnu.2017.01.021 28222963

[27] Hafi E, Soradi R, Diab S, Samara AM, Shakhshir M, Alqub M, et al. Nutritional status and quality of life in diabetic patients on hemodialysis: a cross-sectional study from Palestine. J Health Popul Nutr. 2021;40(1):30. doi: 10.1186/s41043-021-00255-w 34225818 PMC8256194

[28] Hussien H, Apetrii M, Covic A. Health-related quality of life in patients with chronic kidney disease. Expert Rev Pharmacoecon Outcomes Res. 2021;21(1):43–54. doi: 10.1080/14737167.2021.1854091 33213186

[29] Spiegel BMR, Melmed G, Robbins S, Esrailian E. Biomarkers and health-related quality of life in end-stage renal disease: a systematic review. Clin J Am Soc Nephrol. 2008;3(6):1759–68. doi: 10.2215/CJN.00820208 18832106 PMC2572281

[30] Kang SH, Do JY, Kim JC. Impedance-derived phase angle is associated with muscle mass, strength, quality of life, and clinical outcomes in maintenance hemodialysis patients. PLoS One. 2022;17(1):e0261070. doi: 10.1371/journal.pone.0261070 35020730 PMC8754345

[31] Sukackiene D, Rimsevicius L, Miglinas M. Standardized Phase Angle for Predicting Nutritional Status of Hemodialysis Patients in the Early Period After Deceased Donor Kidney Transplantation. Front Nutr. 2022;9:803002. doi: 10.3389/fnut.2022.803002 35252294 PMC8889040

[32] Mundstock E, Amaral MA, Baptista RR, Sarria EE, Dos Santos RRG, Filho AD, et al. Association between phase angle from bioelectrical impedance analysis and level of physical activity: Systematic review and meta-analysis. Clin Nutr. 2019;38(4):1504–10. doi: 10.1016/j.clnu.2018.08.031 30224304

[33] Sember V, Bogataj Š, Ribeiro JC, Paravlić A, Pajek M, Pajek J. Accelerometry Correlates in Body Composition, Physical Fitness, and Disease Symptom Burden: A Pilot Study in End-Stage Renal Disease. Front Physiol. 2021;12:737069. doi: 10.3389/fphys.2021.737069 34950045 PMC8688961

[34] Zhang H, Tao X, Shi L, Jiang N, Yang Y. Evaluation of body composition monitoring for assessment of nutritional status in hemodialysis patients. Ren Fail. 2019;41(1):377–83. doi: 10.1080/0886022X.2019.1608241 31057002 PMC6508072

[35] Ikizler TA, Burrowes JD, Byham-Gray LD, Campbell KL, Carrero J-J, Chan W, et al. KDOQI Clinical Practice Guideline for Nutrition in CKD: 2020 Update. Am J Kidney Dis. 2020;76(3 Suppl 1):S1–107. doi: 10.1053/j.ajkd.2020.05.006 32829751

[36] Leal VO, Stockler-Pinto MB, Farage NE, Aranha LN, Fouque D, Anjos LA, et al. Handgrip strength and its dialysis determinants in hemodialysis patients. Nutrition. 2011;27(11–12):1125–9. doi: 10.1016/j.nut.2010.12.012 21454052

[37] Kim CR, Shin J-H, Hwang JH, Kim SH. Monitoring Volume Status Using Bioelectrical Impedance Analysis in Chronic Hemodialysis Patients. ASAIO J. 2018;64(2):245–52. doi: 10.1097/MAT.0000000000000619 28665828

[38] Yajima T, Yajima K. Association of extracellular water/total body water ratio with protein-energy wasting and mortality in patients on hemodialysis. Sci Rep. 2023;13(1):14257. doi: 10.1038/s41598-023-41131-3 37652929 PMC10471676

[39] Pham MD, Dao TV, Vu ATX, Bui HTQ, Nguyen BT, Nguyen ATT, et al. Association of Bioelectrical Impedance Analysis Parameters with Malnutrition in Patients Undergoing Maintenance Hemodialysis: A Cross-Sectional Study. Medicina (Kaunas). 2025;61(8):1396. doi: 10.3390/medicina61081396 40870441 PMC12388193

